# Uptake of funded genomic testing for syndromic and non-syndromic intellectual disability in Australia

**DOI:** 10.1038/s41431-023-01417-6

**Published:** 2023-07-03

**Authors:** Dylan A. Mordaunt, Kim Dalziel, Ilias Goranitis, Zornitza Stark

**Affiliations:** 1 Health Economics Group, School of Population and Global Health, University of Melbourne, Parkville, VIC Australia; 2 Department of Paediatrics, University of Melbourne, Parkville, VIC Australia; 3 Murdoch Children’s Research Institute, Melbourne, VIC Australia; 4 Australian Genomics, Melbourne, VIC Australia; 5 Victorian Clinical Genetics Services, Murdoch Children’s Research Institute, Melbourne, VIC Australia

**Keywords:** Health policy, Health care economics, Health care, Economics

Lack of reimbursement for genomic testing in rare diseases is recognized as one of the principal barriers to wider implementation within healthcare systems [1]. Multiple studies have provided evidence for diagnostic and clinical utility and for the cost-effectiveness of genomic testing in rare diseases, leading to testing being funded across a range of public and private healthcare systems worldwide [2].

In Australia, healthcare system implementation has been accelerated by many investments at the state and federal levels [3]. Australian data on utility and cost-effectiveness, including long-term patient and family outcomes and benefits of reanalysis, led to an application for public funding in syndromic and non-syndromic intellectual disability [4]. National coverage was approved through the federally-funded Medicare Benefits Scheme (MBS) beginning May 2020, for children under ten years for tests ordered by a paediatrician or clinical geneticist. Paediatricians require patient-specific approval by a clinical geneticist to initiate testing [5]. Interval reanalysis of sequencing data and cascade testing were also funded at this time.

Three years later, we evaluate the uptake, geographical distribution of uptake and cost of MBS-reimbursed genomic testing for syndromic and non-syndromic intellectual disability in Australia and compare this with the predicted utilization used in the health technology assessment and funding process. Actual utilization and reimbursement data were obtained from Medicare Services between May 2020 and April 2023, broken down by state/territory. Predicted utilization estimates were extracted from public summary documents. The budget impact (cost) is reported in Australian Dollars ($, AUD) and Euros (€, EUR) at an actual value in each year reported and by state/territory.

Predicted and actual utilization of services and cost for each test category of initial genomic testing, reanalysis and cascade testing are reported in Table 1 and visualized in Fig. 1. There were 269 initial tests in year 1, 802 in year 2 and 812 in year 3, which were 7.9%, 23.2% and 23.2% of predicted, respectively. Of initial testing during the study period, 17% (320) were singletons, 83% (1563) were trio. The rate of utilization in each state differed considerably (see Table 2).

**Table 1:** Actual and predicted test utilization and reimbursement cost for genomic testing in intellectual disability, by test category and year.

		2020–21	2021–22	2022–23	2023–24	2024–25
Initial Testing						
Predicted	Number of tests	3406	3454	3503	2753	2803
	Cost (AUD)	$6,947,777	$7,046,812	$7,147,009	$5,616,125	$5,718,060
	Cost (EUR)	€4,328,125	€4,389,819	€4,452,236	€3,498,571	€3,562,071
Actual	Number of tests	269	802	812	-	-
	Cost (AUD)	$740,259	$2,148,999	$2,159,403	-	-
	Cost (EUR)	€461,145	€1,338,721	€1,345,202	-	-
First re-analysis						
Predicted	Number of tests	-	-	1448	1468	1488
	Cost (AUD)	-	-	$481,553	$488,417	$495,362
	Cost (EUR)	-	-	€299,984	€304,260	€308,586
Actual	Number of tests	0	1	11	-	-
	Cost (AUD)	$0	$425	$4675	-	-
	Cost (EUR)	€0	€265	€2912	-	-
Single-variant cascade tests						
Predicted	Number of tests	5235	5309	5385	4231	4308
	Cost (AUD)	$1,779,760	$1,805,129	$1,830,796	$1,438,640	$1,464,752
	Cost (EUR)	€1,108,703	€1,124,507	€1,140,496	€896,202	€912,469
Actual	Number of tests	0	2	21	-	-
	Cost (AUD)	$0	$680	$7140	-	-
	Cost (EUR)	€0	€424	€4448	-	-
Total childhood syndrome testing						
Predicted	Number of tests	8641	8763	10,336	8452	8599
	Cost (AUD)	$8,727,537	$8,851,941	$9,459,358	$7,543,182	$7,678,174
	Cost (EUR)	€5,436,828	€5,514,325	€5,892,717	€4,699,033	€4,783,126
Actual	Number of tests	269	805	844	-	-
	Cost (AUD)	$740,259	$2,150,104	$2,171,218	-	-
	Cost (EUR)	€461,145	€1,339,409	€1,352,562	-	-

**Fig. 1:**
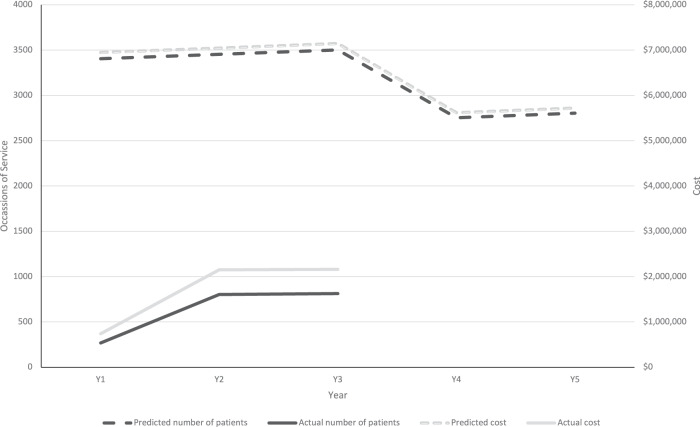
Actual vs predicted services and reimbursement cost for childhood syndrome initial testing. This shows a significant difference between actual and predicted utilisation.

**Table 2:** Rates of testing per 100,000 population, by state and test category.

	State								Total
	NSW	VIC	QLD	SA	WA	TAS	ACT	NT	
Genomic tests	4.97	2.91	1.69	10.49	0.22	4.56	3.08	3.88	3.60
Reanalysis	0.06	0.03	0.00	0.22	0.00	0.00	0.22	0.00	0.05
Cascade tests	0.06	0.01	0.02	0.02	0.00	0.06	0.07	0.00	0.03

Our analysis of actual utilization of funded genomic testing for children with intellectual disability in Australia found uptake to be considerably less than predicted, with a marked geographical variation. These results mirror findings from other healthcare systems reporting underutilization of genomic testing in cancer for example [6]. Possible contributors to the disparity between actual and predicted uptake include the accuracy of the initial population estimates and corresponding predictions as well as factors contributing to underutilization at patient, clinician and system levels.

The initial population estimates were developed by the applicant by incorporating observed local (Victorian) testing levels as well as expert advice. Through the application process, a number of issues were raised around these estimates [5]. There was a significant level of uncertainty about the predicted utilization and ultimately actual utilization was much lower than predicted and closer to the applicant’s original estimates [5]. These results demonstrate the difficulty of predicting genomic testing utilization given the complexity both of estimating cohort size combined with determinants of implementation (and resulting predictors of utilization).

A recent systematic review identified a broad range of determinants of utilization, including service-level factors, professional attitudes and values, training and workforce needs, as well as patient factors and public perceptions [7]. Many of these are applicable across different healthcare systems with health professionals consistently expressing positive attitudes and beliefs towards genomic testing [8], but also consistently reporting being underprepared for genomic medicine. From a practical perspective, complex logistics in receiving approvals for testing are a known barrier to uptake [8]. Of note, the reimbursement mechanism deployed in Australia includes a dependency on specialist clinical geneticists to either order the test or to provide approval to paediatricians on a case-by-case basis. However, there is currently no reimbursement mechanism for clinical geneticist’s time to provide this support nor to support paediatrician test use through genomic multi-disciplinary team meetings, which have proven effective in supporting mainstreaming of genomic testing in many areas.

We observed marked variability in geographic access to genomic testing in Australia, despite the presence of a national funding mechanism. This variability doesn’t differentiate causation. All jurisdictions in Australia have public clinical genetics services. However, these are configured and resourced differently, which may contribute to variability in access to testing. For instance, most clinical genetics services are situated in metropolitan centres, so patients and non-genetic clinicians in rural and regional areas may have less access to genomic testing or specialist support [9]. Access issues are particularly prominent for Indigenous populations [10]. Not all jurisdictions in Australia have local access to genomic testing laboratories. Although testing can be arranged by sending specimens to inter-state laboratories, exposure to laboratory staff and testing may be an important factor in uptake. Lastly, unequal investment in initiatives designed to build infrastructure, workforce capacity and capability at the state level may have also influenced uptake [4].

A number of policy variations to improve utilization could therefore be considered, including reducing constraints on test ordering (e.g. allowing paediatricians to order based on demonstrating competence and/or removing age limits on eligibility), providing a reimbursement mechanism for clinical geneticist’s and genetic counsellor’s time to support broader uptake, and investing nationally in workforce and digital support infrastructure.

Our study did not capture tests performed in individuals with intellectual disability during the study period funded through alternative mechanisms, such as hospital budgets, research studies or families. It is worth noting that the public funding came into effect in the early months of the COVID-19 pandemic and reduced operation of ambulatory clinics and testing is also likely to have contributed to these observations. However, results based on nationally funded tests are likely to capture the majority of use.

In conclusion, the observation of apparent test underutilization despite national coverage in Australia underscores the importance of coordinated efforts to promote the uptake of genomic testing in order to improve overall outcomes for patients and families with rare disease. Ongoing monitoring of test utilization will be an important measure of the success of future interventions.

## Data Availability

Data is readily available from Medicare Services for utilization in aggregated form.

## References

[1] Stark Z, Dolman L, Manolio TA, Ozenberger B, Hill SL, Caulfied MJ (2019). Integrating Genomics into Healthcare: A Global Responsibility. Am J Hum Genet.

[2] Phillips KA, Douglas MP, Wordsworth S, Buchanan J, Marshall DA. Availability and funding of clinical genomic sequencing globally. BMJ Glob Health. 2021;6:1–8. 10.1136/bmjgh-2020-004415 PMC7880109 33574068

[3] Stark Z, Boughtwood T, Haas M, Braithwaite J, Gaff CL, Goranitis I (2023). Australian Genomics: Outcomes of a 5-year national program to accelerate the integration of genomics in healthcare. Am J Hum Genet.

[4] Stark Z, Schofield D, Alam K, Wilson W, Mupfeki N, Macciocca I (2017). Prospective comparison of the cost-effectiveness of clinical whole-exome sequencing with that of usual care overwhelmingly supports early use and reimbursement. Genet Med.

[5] Schofield D, Rynehart L, Shresthra R, White SM, Stark Z (2019). Long-term economic impacts of exome sequencing for suspected monogenic disorders: diagnosis, management, and reproductive outcomes. Genet Med.

[6] Schilsky RL, Longo DL (2022). Closing the Gap in Cancer Genomic Testing. N. Engl J Med.

[7] Pearce C, Goettke E, Hallowell N, McCormack P, Flinter F, McKevitt C (2019). Delivering genomic medicine in the United Kingdom National Health Service: a systematic review and narrative synthesis. Genet Med.

[8] Hamilton AB, Oishi S, Yano EM, Gammage CE, Marshall NJ, Scheuner MT (2014). Factors influencing organizational adoption and implementation of clinical genetic services. Genet Med.

[9] Nisselle A, King EA, McClaren B, Janinski M, Metcalfe S, Gaff C (2021). Measuring physician practice, preparedness and preferences for genomic medicine: a national survey. BMJ Open.

[10] Luke J, Dalach P, Tuer L, Savarirayan R, Ferdinand A, McGaughran J (2022). Investigating disparity in access to Australian clinical genetic health services for Aboriginal and Torres Strait Islander people. Nat Commun.

